# Generation of Oligodendrocyte Progenitor Cells From Mouse Bone Marrow Cells

**DOI:** 10.3389/fncel.2019.00247

**Published:** 2019-06-05

**Authors:** Yuan Zhang, Xin-Yu Lu, Giacomo Casella, Jing Tian, Ze-Qing Ye, Ting Yang, Juan-Juan Han, Ling-Yu Jia, Abdolmohamad Rostami, Xing Li

**Affiliations:** ^1^The Key Laboratory of Medicinal Resources and Natural Pharmaceutical Chemistry, National Engineering Laboratory for Resource Development of Endangered Crude Drugs in Northwest China, The Ministry of Education, College of Life Sciences, Shaanxi Normal University, Xi’an, China; ^2^Department of Neurology, Thomas Jefferson University, Philadelphia, PA, United States

**Keywords:** oligodendrocyte progenitor cells, oligodendrocytes, neurospheres, bone marrow, autologous cells

## Abstract

Oligodendrocyte progenitor cells (OPCs) are a subtype of glial cells responsible for myelin regeneration. Oligodendrocytes (OLGs) originate from OPCs and are the myelinating cells in the central nervous system (CNS). OLGs play an important role in the context of lesions in which myelin loss occurs. Even though many protocols for isolating OPCs have been published, their cellular yield remains a limit for clinical application. The protocol proposed here is novel and has practical value; in fact, OPCs can be generated from a source of autologous cells without gene manipulation. Our method represents a rapid, and high-efficiency differentiation protocol for generating mouse OLGs from bone marrow-derived cells using growth-factor defined media. With this protocol, it is possible to obtain mature OLGs in 7–8 weeks. Within 2–3 weeks from bone marrow (BM) isolation, after neurospheres formed, the cells differentiate into Nestin^+^ Sox2^+^ neural stem cells (NSCs), around 30 days. OPCs specific markers start to be expressed around day 38, followed by RIP^+^O4^+^ around day 42. CNPase^+^ mature OLGs are finally obtained around 7–8 weeks. Further, bone marrow-derived OPCs exhibited therapeutic effect in shiverer (Shi) mice, promoting myelin regeneration and reducing the tremor. Here, we propose a method by which OLGs can be generated starting from BM cells and have similar abilities to subventricular zone (SVZ)-derived cells. This protocol significantly decreases the timing and costs of the OLGs differentiation within 2 months of culture.

## Introduction

Oligodendrocyte progenitor cells (OPCs) are present in both the white and gray matter of the adult central nervous system (CNS); upon oligodendrocytes (OLGs) injury, OPCs contribute to OLGs regeneration and remyelination (Duncan et al., 2018). OPCs are characterized by expression of platelet-derived growth factor receptor alpha (PDGFRα), neural/glial antigen 2 (NG2), and A2B5 (Dietrich et al., 2002; Zhu et al., 2008; Sim et al., 2011); they represent a high proliferative cell population resident in the CNS of adult mammals and humans (Mekhail et al., 2012; Clemente et al., 2013). OLGs derived from OPCs and are the myelinating cells of the CNS. Once OPCs differentiate into mature OLGs, they extend multiple processes that individually ensheath axons and then proceed to generate the concentric layers of the modified cell membrane that compose myelin (Goldman and Kuypers, 2015). Thanks to their remyelinating ability, several groups used OPCs transplantation as a therapeutic strategy for CNS diseases (Kim et al., 2012; Najm et al., 2013; Yang et al., 2013). Indeed, OPCs can be used as a safe treatment for spinal cord injury (Nori et al., 2018), stroke (Wang et al., 2018), Parkinson’s disease (Ahmed et al., 2013), and cerebral palsy (Rumajogee et al., 2018).

The transplantation of exogenous OPCs can represent a suitable treatment for chronic demyelinating disorders (Czepiel et al., 2015). The most common methods for obtaining functional OPCs, use mouse embryonic stem cell (ESCs) or pluripotent stem cells (Li et al., 2013; Wang et al., 2013; Kuai et al., 2015; Manley et al., 2017), or reprogrammed fibroblasts expressing a defined set of transcription factors (TFs) (Najm et al., 2013; Yang et al., 2013; Yamashita et al., 2017). Even if, OPCs transplantation has shown promising result in rodents (Goldman and Kuypers, 2015), its application remains far from the clinic. Several barriers should be overcome such as, whether OPCs can successfully migrate to the lesion (Foster et al., 1995), or their reprogramming can induce aberrant phenotype with consequent side-effects, as reported in some study in which next-generation sequencing was used (Liu et al., 2019). Other well-reported approaches used SVZ, from rodent CNS, as a source for isolating functional OPCs (Dincman et al., 2012; Zhu et al., 2014; Lu et al., 2015); however, due to the low cellular yield these methods have not guaranteed any feasibility for clinical application (Yang et al., 2010). Recently, it has been demonstrated that OPCs can be generated from bone marrow (BM) (Cristofanilli et al., 2011). These bone marrow-derived OPCs, induced with specific growth factor media, have shown similar morphology and cellular markers to canonical OPCs (Nazm Bojnordi et al., 2015).

We describe here a detailed protocol for generating OPCs and mature OLGs from autologous mouse bone marrow-derived neural stem cells (NSCs). Our method consists of two main steps. The first step provides the differentiation and generation of neurospheres from bone marrow-derived NSCs; the second one consists of neurospheres-derived OPCs differentiation within 1 week, using specific media. Respect to a previous report (Douvaras and Fossati, 2015), our protocol enables to produce autologous OLGs in 52 days.

Obtaining a high number of autologous OLGs is relevant to the clinic. For addressing this aspect, we tested the myelination ability of our bone marrow-derived OPCs, in a genetic model of congenital dysmyelination, shiverer (Shi) mice. Bone marrow-derived OPCs induced remyelination and significantly reduced tremor in treated mice. According to our data, this protocol is suitable for producing in large scale autologous OPCs, research aimed at understanding oligodendrocyte biology, and finally, it may represent a screening platform for myelinating compounds.

## Materials and Methods

### Animals

C57BL/6 (The Fourth Military Medical University), SJL, and C3H mouse strains (The Jackson laboratory) are all appropriate for this protocol. Mice were kept in clean cages with a maximum of 5 mice per cage, in a controlled environment with 12/12 h of light/dark cycles and food *ad libitum* throughout the experimental procedures. All experimental procedures and protocols are approved by the Animal Management and Committee of Shaanxi Normal University. At the same time, by the approved institutional guidelines and regulations.

BM-NSCs were given by injection of single cells suspension (2 × 10^5^ cells in 20 μl PBS/each mouse) by intracerebroventricular (i.c.v.) injection, into 1 week-old Shi mice. PBS-treated age-sex- and strain-matched mice were used as controls. All groups of animals were observed for 35 days.

### Preparation of Neural Stem Cell Proliferation Media (NSC-PM)

A total of 200 ml of NSC-PM media is prepared mixing 150 ml of DMEM/F12 with 4 ml of B27, 2 ml of Penicillin-streptomycin (50 U/ml), 2 ml of Non-essential amino acid solution (0.1 mM), 2 ml HEPES (10 mM), 2 ml of sodium pyruvate (1 mM; all purchased from Thermo Fisher Scientific), 40 μl of epidermal growth factor (EGF, 20 ng/ml, stock concentration: 100 μg/ml; PeproTech), and 40 μl of basic fibroblast growth factor (bFGF, 10 ng/ml, stock concentration: 50 μg/ml; PeproTech). DMEM/F12 is added to a final volume of 200 ml, filter with a bottle-top filter (0.22 μm) and store at 4°C. We suggest to use complete media within 2 weeks.

### Preparation of OPC Proliferation Media (OPC-PM)

A total of 50 ml of OPC-PM is prepared mixing 30 ml of DMEM/F12 with 1 ml of B27, 0.5 ml of N2, 0.5 ml of GlutaMax (2 mM), 0.5 ml of Penicillin-streptomycin (50 U/ml), 0.5 ml of Non-essential amino acid solution (0.1 mM), 0.5 ml of sodium pyruvate (1 mM; all purchased from Thermo Fisher Scientific), 10 μl of platelet-derived growth factor-AA (PDGF-AA, 20 ng/ml, stock concentration: 100 μg/ml; PeproTech), and 20 μl of bFGF (20 ng/ml, stock concentration: 50 μg/ml; PeproTech). DMEM/F12 is added to a final volume of 50 ml, filter with a bottle-top filter (0.22 μm) and store at 4°C. We suggest to use complete media within 2 weeks.

### Preparation of Early OLGs Differentiation Media (EOLG-DM)

A total of 25 ml of EOLG-DM is prepared mixing 15 ml of DMEM/F12 with 0.5 ml of B27, 0.25 ml of N2, 0.25 ml of GlutaMax (2 mM), 0.25 ml of Penicillin-streptomycin (50 U/ml), 0.25 ml of Non-essential amino acid solution (0.1 mM), 0.25 ml of sodium pyruvate (1 mM; all purchased from Thermo Fisher Scientific), 50 μl of triiodo-l-thyronine (T3, 40 ng/ml, stock concentration: 20 μg/ml; Sigma-Aldrich), 20 μl of sonic hedgehog (Shh) (40 ng/ml stock, concentration: 50 μg/ml), 20 μl of Noggin (40 ng/ml, stock concentration: 50 μg/ml), 5 μl of insulin-like growth factor (IGF, 100 ng/ml, stock concentration: 500 μg/ml; all purchased from PeproTech), and 25 μl of neurotrophin 3 (NT-3, 10 ng/ml; Sigma-Aldrich). DMEM/F12 is added to a final volume of 25 ml, filter with a bottle-top filter (0.22 μm) and store at 4°C. We suggest to use complete media within 1 week.

### Preparation of Late OLGs Differentiation Media (LOLG-DM)

A total of 10 ml of LOLG-DM is prepared mixing 10 ml of EOLG-DM with 5 μl of 3′,5′-cyclic adenosine monophosphate (cAMP, 50 μM, stock concentration: 50 μg/ml; Sigma-Aldrich). The fresh media should be using within 1 week. For preparing Poly-D-lysine coating solution, stock at 0.1 % (wt/vol) 50 mL of sterile tissue culture grade water to 5 mg of poly-D-lysine (Sigma-Aldrich). Poly-D-lysine solution can be stored at 4°C for 3 months. Coating culture plates with the poly-D-lysine coating solution (0.5 ml per well for 24 well plate) and keep them for 2 h in 37°C incubator or overnight at 4°C. Remove coating solution and wash three times with sterile ddH_2_O. After poly-D-lysine coated, add 1 μg/ml laminin (Sigma-Aldrich) solution for 2 h in 37°C incubator or overnight at 4°C. Laminin should be slowly thawed at 2–8°C for avoiding any solidification process. Dilute in a balanced salt solution and coat culture surface with a minimal volume. Remove coating solution, wash three times with sterile ddH_2_O and dry completely in a tissue culture hood. Allow to air dry at least 5 min before introducing cells and media. Poly-D-lysine and laminin-coated coverslips: Place sterilized coverslips into the wells of a 24 well plate. The procedure followed the Poly-D-lysine coating and Laminin coating. The coverslips will float on the surface of the solution. Make sure the coverslips are completely immersed in the coating buffer to ensure the coverslips are well coated.

### Isolation and Culture of NSCs From Murine SVZ and Bone Marrow

SVZ-derived NSCs were isolated from adult C57BL/6 mice as described previously with minor modification (Yang et al., 2009). Briefly, C57BL/6 mice 8 weeks old were euthanized and the SVZ region was harvested under sterile conditions and placed in DMEM media. After a brief washing with DMEM media, tissues were cut into 1 mm^3^ pieces and digested by neural tissue dissociation kits (Miltenyi Biotec). The cells were suspended in serum-free DMEM/F12 (Invitrogen) supplied with 2% B27 supplements (Invitrogen), 20 ng/ml EGF (Peprotech) and 10 ng/ml bFGF (Peprotech), along with 100 IU/ml penicillin and 100 μg/ml Streptomycin (Sigma).

Bone marrow cells were isolated according to a previously described protocol (Swamydas and Lionakis, 2013). Finally, the cells were suspended in NSC-PM media at 10^6^ cells/ml, and plated on poly-D-lysine, and laminin coated 24 well plate for 4 days. We suggest to replace half of the media with fresh NSC-PM media every 4 days. The media should be changed softly and slowly to avoid lifting the adhered NSCs (This is the critical step).

The formation of cellular clusters that resemble neurospheres becomes evident after 10–14 days. For cell detachment, aspirate the media, add 500 μl accutase (Thermo Fisher Scientific), and incubate at room temperature (22–25°C) for 2 min. Use 200 μl pipette to carefully suspend the adhered cell and transfer to a 15 ml conical tube. Shake the tube 10 times/min for total 3 min and add 4.5 ml DPBS for centrifuge (300 ×*g*, 10 min). Next, suspend the cell pellet in NSC-PM media at 1 × 10^5^ cells/ml, and plate the cell in non-coated 6 cm plate. After 4 days, the neurospheres can observe under the microscope. Collect suspended neurospheres from culture dish and transfer to a 15 ml conical tube. Centrifuge the cell at 100 ×*g* for 1 min. Gently aspirate media leaving the neurospheres at the bottom of tube. Resuspend neurospheres in 5 ml DPBS and centrifuge the cell at 100 × g for 1 min to remove the media residues. Gently aspirate media leaving the neurospheres at the bottom of tube and add 1 ml of accutase to the cell culture and incubate 5 min at room temperature. Mildly shake the tube 10 times/min. If any neurosphere remains visible, after 4 min accutase incubation, we suggest to pipette gentle up and down until all the visible neurospheres are in a single cell suspension. The accutase media will change the color from carnation to oyster white. Then, add 4 ml of fresh media to the tube and centrifuge the cells at 200 ×*g* for 4 min. Gently aspirate the supernatant and resuspend cells in fresh media. Transfer the cell 1 × 10^5^ cells/ml to a new culture dish and incubate at 37°C for NSCs expanded. Cells can be frozen at this step. For visualization of results, seed the cell at 1 × 10^5^ cells/ml in a poly-D-lysine and laminin coated 24 well plate with coverslips. Cells were fixed using 4% paraformaldehyde for immunocytochemical staining.

### Bone Marrow-Derived NSCs Differentiation and Proliferation

To test the proliferation capacity of bone marrow-derived NSCs, growth curve of bone marrow-derived NSCs was determined followed previous method (Yang et al., 2010). Briefly, the newly formed neurospheres were digested to the single cells and plated at a density of 1.0 × 10^5^ cell/ml and cultured in NSC-PM. At days 6, 11, 16 and 21, neurospheres in each well were digested into single cells; cell numbers were counted by hemocytometer. SVZ derived NSCs were used in the parallel experiment to compare the proliferate ratio.

To evaluate the differentiation ability of bone marrow-derived NSCs, single cells were plated on poly-D-lysine/laminin coated coverslip at a density of 1.0 × 10^4^ cells/ml and cultured in specific NSCs differentiation media (Li et al., 2016). In brief, for neuron differentiation, Neurobasal media was supplemented with 2% B27, 2 mM GlutaMax-I and 0.5 mM cAMP. For astrocyte differentiation, DMEM was supplemented with 1% N2, 2 mM GlutaMax-I and 1% FBS. The oligodendrocyte differentiation media requires Neurobasal media supplemented with 2% B27, 2 mM GlutaMax-I and 20 ng/ml T3. Over 2 weeks, NSCs in differentiation media changed morphology and developed markers of neurons, astrocytes, and OLGs as determined by immunocytochemistry staining. To determine the number of cells expressing a specific antigen, five areas of each coverslip were examined, and the percentage of positive cells labeled for a specific neural marker in the total number of DAPI^+^ cells was expressed as the mean value of specific neural differentiation.

### OPCs Differentiation

Once neurospheres reach 100–250 μm of diameter, dissociate the neurospheres with accutase. Start by stepping suspend the cell pellet in NSC-PM media at 1 × 10^5^ cells/ml until transfer the cell 1 × 10^5^ cells/ml to a new culture dish and incubate at 37°C for NSCs expanded to the end. Finally, The cells are seeded on the poly-D-lysine, and laminin coated plate at 5 × 10^3^ cells/ml in the NSC-PM media. After 1–2 days, over 90% cells will be adhered to the bottom, change the media to OPC-PM. Incubate the plate in a 37°C, 5% CO_2_ incubator and perform media changes every 2 days for 8 days. Cells can be frozen at this step.

### OLGs Maturation

At day 9, aspirate the OPC-PM media, gently rinse with DPBS to remove the growth factor (i.e., PDGF-AA and bFGF), and add fresh EOLG-DM (500 μl/well/24 well plate) for inducing differentiation of OLGs. OPCs will proliferate few times. If the cells reach 90% confluency, it might be necessary to split the cells at a 1:4–6 ratio. After 4 days, the media is changed to LOLG-DM. Adding cAMP will accelerate the branch outgrowth for OLGs differentiation. Repeat these steps for 3 times, after 2 weeks.

### Immunofluorescence Staining

For immunocytochemistry, cells were fixed in 4% paraformaldehyde at room temperature for 30 min. Then, incubate with 0.3% TritonX-100 (in PBS) for 15 min at room temperature and wash with PBS. The primary antibody is diluted to a suitable concentration with a blocking solution (10% horse serum in PBS). The following primary antibodies were used: anti-A2B5 (MA1-90445, Thermo Fisher Scientific), anti-CNPase (ab44289, Abcam), anti-Ki67 (ab15580, Abcam), anti-Nestin (MAB353, Milipore), anti-NG2 (MAB5384, Milipore), anti-O4 (NL1326V, R&D), anti-RIP (ab72139, Abcam), and anti-SOX2 (ab97959, Abcam). Primary antibodies were washed out with PBS three times after overnight incubation at 4°C. The corresponding Alexa Fluor 488/594 conjugated secondary antibodies (both from Jackson ImmunoResearch Laboratories) were used for 2 h at room temperature. After wash, the ProLong^TM^ Gold Antifade Mountant with DAPI buffer (P36931, Thermo Fisher Scientific) were used for mounting slides. Results were visualized by fluorescent microscopy (Nikon Eclipse E600; Nikon, Melville, NY, United States). Quantitative image analysis was performed using ImagePro (Media Cybernetics).

### Bone Marrow NSC-Derived OPCs Transplantation Into Shi Mice

Newborn double-homozygous Shi (*Mbp^Shi^/Mbp^Shi^*) mice (The Jackson Laboratory, Bar Harbor, ME, United States) were injected with dissociated single OPCs. The protocol followed previous work with minor modifications (Zhang et al., 2019). Briefly, to generate the lentivirus expression of mCherry protein, the backbone of pCDH-CMV-MCS-EF1-copGFP (System Biosciences) was used. Subsequently, the copGFP was replaced by mCherry and puromycin was inserted into the multiple clone sites. The positive plasmid was confirmed by sequencing and using for lentivirus packaging. OPCs were transfected with lentiviral expressed mcherry, and positive cells were selected by puromycin. Then, mcherry OPCs^+^ were transplanted bilaterally in the corpus callosum of 1 week-old Shi mice (2.0 × 10^5^ cell/mice). PBS-treated age-sex-, strain-matched mice, and sham control mice were used. After 5 weeks from the treatment, mice were anesthetized, and brains were isolated and fixed with 4% paraformaldehyde and cryoprotected using 30% sucrose. CNS coronal sections were stained with myelin basic protein (MBP) antibody (ab40390, Abcam) for evaluating the differentiation process of mcherry OPCs^+^. Tremor (Trembling time/total time) was analyzed for evaluating disease development. Kaplan-Meier analysis was used to assess the survival rate as described (Shinkai et al., 1992).

### Statistical Analysis

Statistical analyses were performed using GraphPad Prism software (GraphPad, La Jolla, CA, United States). Data are presented as mean ± SD. Experiments with 2 groups were tested for statistical significance using unpaired, 2-tailed Student’s *t*-tests. When comparing two groups at different time points, data were analyzed by two-way analysis of variance (ANOVA) with Tukey’s multiple comparisons test. Comparisons between two groups were carried out with Student’s *t*-test. Values of *p* < 0.05 were considered significant.

## Results

### Timeline of Bone Marrow-Derived Mature Oligodendrocyte Differentiation

**FIGURE 1:**
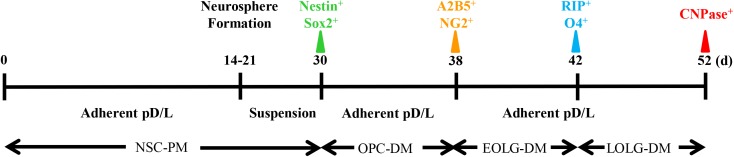
Timeline of oligodendrocyte differentiation. Multicolor triangles represent recommended time points for evaluating the expression of stage-specific markers through immunofluorescence.

The timeline of our protocol is shown in Figure 1. In the first step, NSCs are differentiated from BM cells. After BM isolation, the cells are seeded at high density (5 × 10^5^ cell per cm^2^) on poly-D-lysine/laminin coated 24-well plate. *In vitro*, the presence EGF and fibroblast growth factor-2 (FGF2) mimic the NSCs culture environment, inducing NSCs proliferation. In the second stage, NSCs are induced to become OPCs. Here, the adherent cultures are dissociated to form spheres in suspension. Although Sox2^+^Nestin^+^ cells can also form spheres, the presence of recombinant protein PDGFα will gradually enrich the NG2^+^A2B5^+^ cell population (Neri et al., 2010). The final stage is the transition from OPCs to mature OLGs, by exposing the cells to T3, NT-3, Noggin, Shh, and IGF (Najm et al., 2013; Douvaras and Fossati, 2015).

### Neurosphere Differentiation Step

**FIGURE 2:**
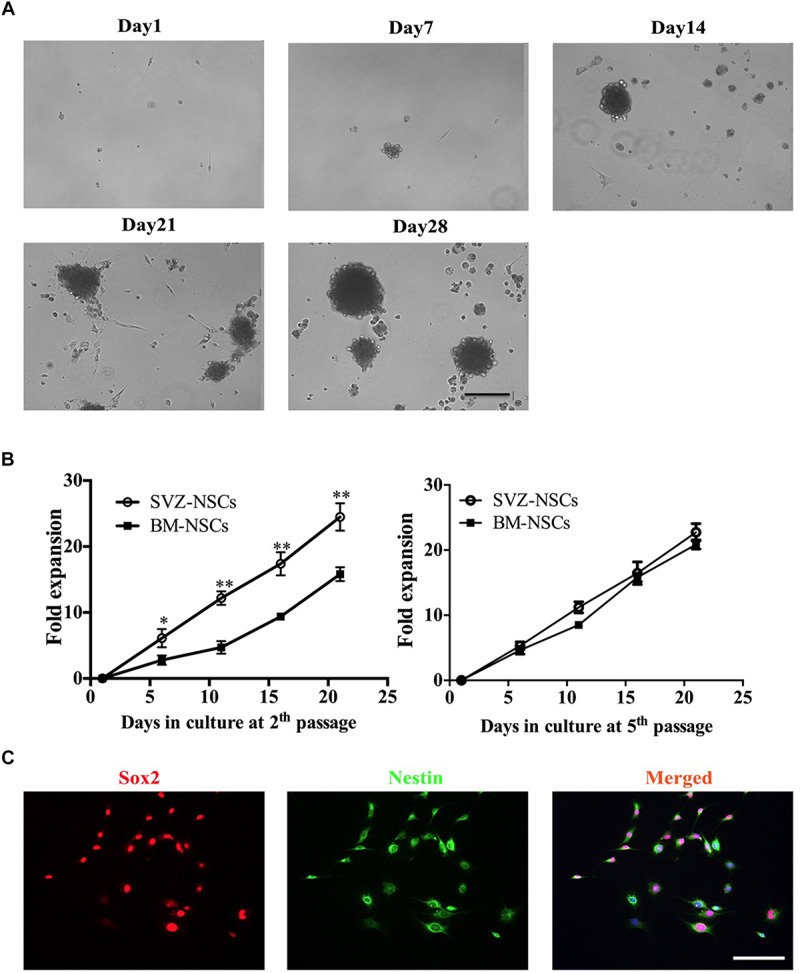
Generation of bone marrow-derived NSCs *in vitro*. BM-NSCs were isolated and expanded from bone marrow of adult C57BL/6 mice and cultured in DMEM/F12 containing 20 ng/ml EGF, 20 ng/ml Bfgf, and 2% B27 supplements. **(A)** Single cells were seeded in poly-D-lysine- and laminin-coated plates. After 7 days, the cells showed obvious proliferation. After 14 days, the NSCs gradually formed neurospheres. After 21–28 days, neurospheres increased size, and detached from the bottom of the culture plate. **(B)** Growth curves of BM-NSCs and SVZ-NSCs. Single cells at the second and fifth passages were seeded at a density of 1.0 × 10^5^ cells/ml and cultured in proliferation media. At days 6, 11, 16 and 21, neurospheres in each well were digested into single cells; cell numbers were counted by hemocytometer. At least 5 wells were evaluated at each time point. Data represent the mean ± SD from three repeated experiments from separately generated cultures, ^∗^*p* < 0.05, ^∗∗^*p* < 0.01, as determined by two-way ANOVA with Tukey’s multiple comparison test. **(C)** Neurospheres dissociated with accutase and plated in the coated coverslips were immunostained for NSC markers Sox2 and Nestin. Scale bar = 50 μm.

For NSCs generation, BM from autologous adult mice can efficiency generate neurospheres. During neurosphere differentiation step, we are usually able to observe three stages (Figure 2A). (1) After 1 week in culture, individual cells exhibit high proliferative ability. (2) Two weeks later cells form neurospheres. (3) At 3–4 weeks, the neurospheres increased in size and gradually detached from the bottom of the culture plate. To characterize the bone marrow-derived NSCs, we compared the proliferative capacity with the SVZ-derived NSCs. We collected the neurosphere, dissociated, and re-plated at 1.0 × 10^5^ cell/ml for a next round cell expansion. We next compared the proliferation capacity of bone marrow- and SVZ-NSCs. BM and SVZ-NSCs from passages 2 and 5 were seeded and cultured in NSC-PM. At day 6, 11, 16, and 21, the neurospheres formed and re-dissociated to single cells, and the cell number were counted by hemocytometer. The proliferation rate of BM-NSCs was significantly slower than SVZ-NSC at the early stage, whereas there was no obviously difference in proliferation capacity at passage 5 (Figure 2B). To identify the BM-NSCs, single cells were transferred onto poly-D-lysine and laminin pre-coated coverslips and used for immunostaining with Sox2 and Nestin. We can observe over 95% cells are Sox2 and Nestin-positive cells at passage 5 (Figure 2C).

### Differentiation Potential of BM-NSCs

**FIGURE 3:**
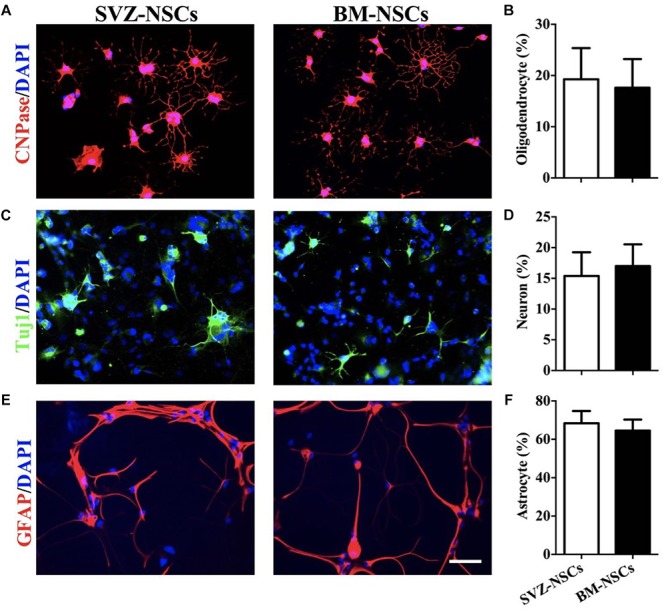
BM-NSCs differentiation *in vitro*. Examples of NSCs that differentiated into **(A)** OLGs (CNPase^+^), **(C)** neuron (Tuj1^+^), and **(E)** astrocytes (GFAP^+^), after 1 week with specific differentiation media. Scale bar = 50 μm. **(B**,**D**,**F)** Quantitative analysis of differentiated cells. Data are shown as mean values ± SD (*n* = 5 each group) and are representative of three experiments. Significance difference was analyzed by Student’s *t*-test.

To test the differentiation potential of BM-NSCs *in vitro*, neurospheres were used and cultured in specific neuron/astrocyte/oligodendrocyte differentiation media. After 2 weeks, BM-NSCs, plated in presence of differentiation media, changed morphology and developed markers of neuron, astrocytes, and OLGs, as shown by immunocytochemistry staining. We compared the differentiation capacity of BM-NSCs and SVZ-NSCs. There are no significant difference about the percentage of CNPase^+^, Tuj1^+^, and GFAP^+^ cells between BM-NSCs and SVZ-NSCs. These results indicated that BM-NSCs can differentiate into neural cell lineages (Figure 3).

### OPCs Differentiation and Oligodendrocyte Maturation Process

**FIGURE 4:**
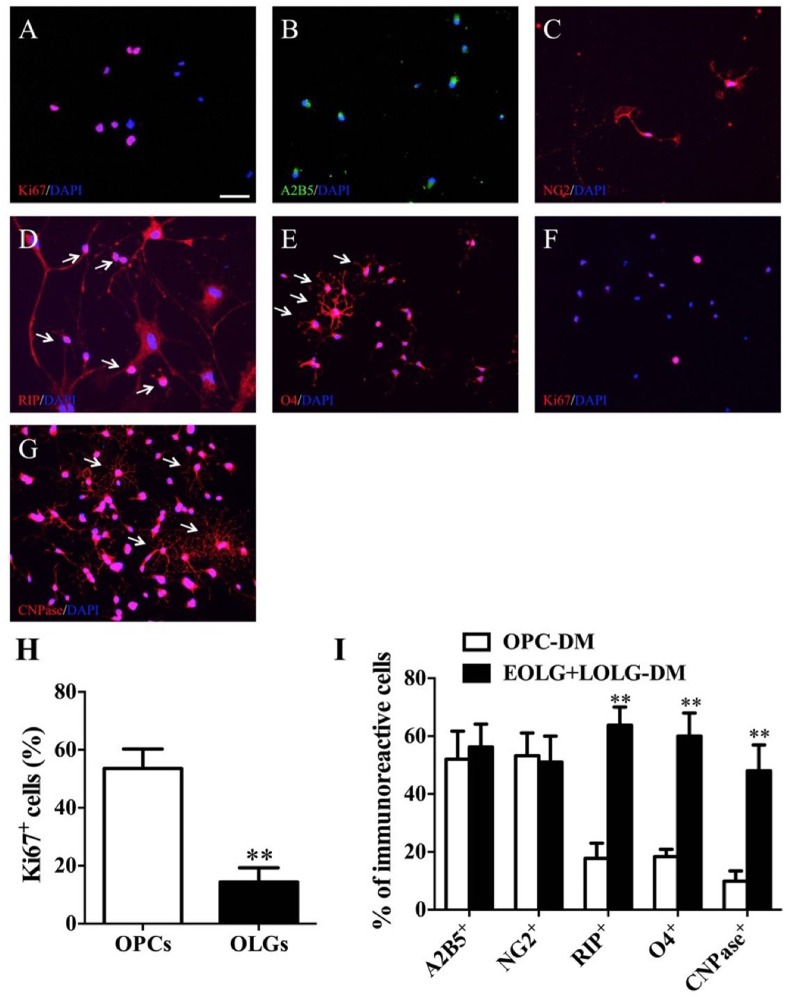
Fundamental steps of BM-NSCs OLGs differentiation. **(A)** Expression of Ki-67 at day 38, as shown by immunofluorescence analysis. **(B**,**C)** Immunofluorescence staining of progenitor cells at day 38, expressing A2B5, and NG2. **(D**,**E)** RIP and O4 staining show cells ramified morphology. **(F)** Expression of Ki-67 which in late stage of OLGs differentiation (day 52). **(G)** At the end of differentiation stage, CNPase^+^ OLGs show typical ramified morphology. **(H)** OPCs and OLGs proliferation **(A**,**F)** was quantified as percentage of Ki67-positive cells. Scale bar = 50 μm. **(I)** The percentages of A2B5^+^, NG2^+^, RIP^+^, O4^+^, and CNPase^+^ cells are present in EOLG+LOLG-DM cultures as compared to OPC-PM cultures. Data are expressed as the mean ± SD, three independent experiments, ^∗∗^*p* < 0.01, Student’s *t*-test.

To assess whether the enrichment of OPCs obtained by differentiation media could be exploited for increasing the yield of mature OLGs, OPCs grown in EOLG-DM media for 4 days were shift to LOLG-DM media for additional 10 days. Cells grown for 14 days in OPC-PM media were used as control. Cells were then analyzed for the presence of A2B5^+^, NG2^+^, RIP^+^, O4^+^, and CNPase^+^ cells. For OPCs differentiation, single NSCs were seed on poly-D-lysine, and laminin coated plates or coverslips in the OPCs differentiation media. Cells exhibited strong proliferation ability and grew faster under the proliferation condition (Figure 4A). These OPCs expressed A2B5 and NG2, as shown by immunostaining (Figure 4B,C). Moreover, OPC-spheres can be cultured with OPCs media without differentiation. They can further form immature OLGs (e.g., RIP^+^ and O4^+^) when are grown in OLGs differentiation media for 1 week (Figure 4D,E).

For OLGs maturation, OPCs were grown in presence of oligodendrocyte differentiation media. Cells gradually stopped to proliferate (Ki67^+^) (Figure 4F,H) and started to differentiate into mature OLGs (CNPase^+^). About 50 % of the CNPase^+^ OLGs exhibited complex membrane morphology indicating OPCs differentiation into mature OLGs successfully (Figure 4G,I).

### OPCs and Mature OLGs Generation From Autologous Mouse Bone Marrow-Derived NSCs

**FIGURE 5:**
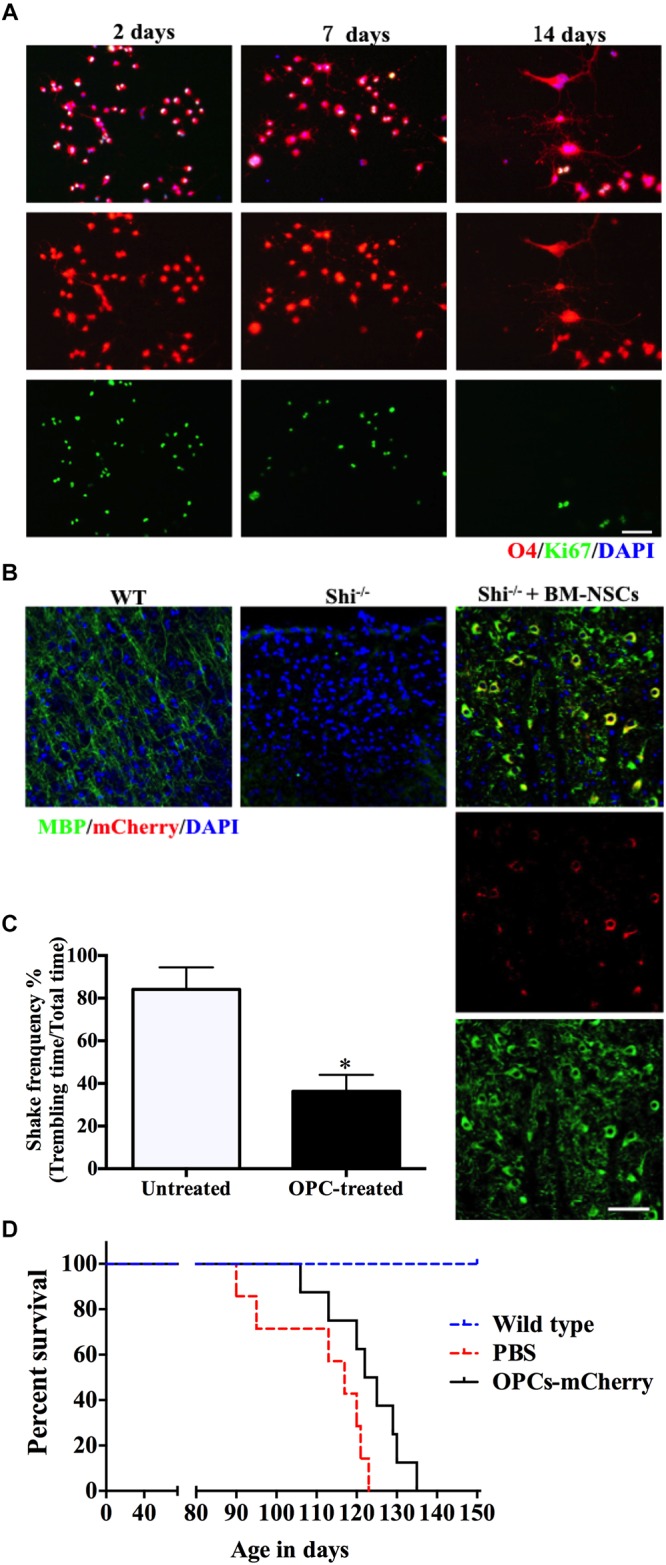
BM-NSCs OLGs differentiation *in vitro* and transplantation in Shi mice. **(A)** BM-NSCs were cultured in oligodendrocyte differentiation media for 48 h, 1 week, and 2 weeks for O4 (differentiation marker) and Ki-67 (proliferation marker) staining. **(B)** Fluorescence staining of BM-NSCs (mCherry^+^ cells), after 35 days post injection, show myelination (MBP^+^) in the contralateral striatum. Scale bar = 50 μm. **(C)** Quantification of tremor of Shi mice treated with OPCs. **(D)** Transplanted OPCs prolong the survival of Shi mice compared with PBS-treated (*n* = 9). Symbols represent mean ± SD. ^∗^*p* < 0.05, Student’s *t*-test.

**FIGURE 6:**
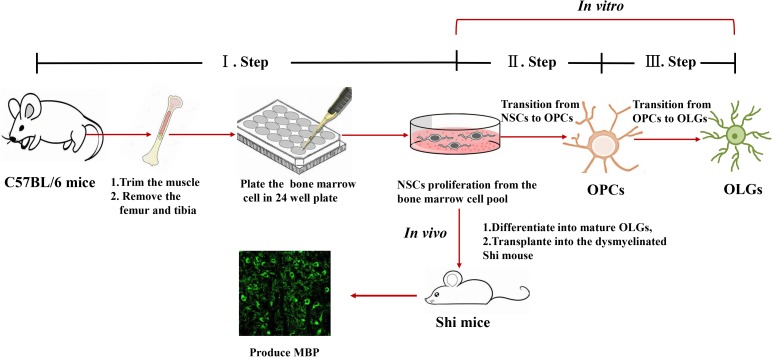
Representative scheme of the protocol for generating OPCs and OLGs from autologous mouse bone marrow-derived NSCs.

Our method allows to isolate OPCs, using a selective detachment procedure, generate mature OLGs *in vitro* (Figure 5A), and induce remyelination after transplantation into the dysmyelinated Shi mice (Figure 5B). Although immunofluorescence analysis showed abundant MBP^+^mCherry^+^ cells, the transplanted cells were more like “non-completedly myelinating” cells. We hypothesized that these cells would become mature at later time points and express more MBP^+^ myelin internodes. To define whether our BM-NSCs had the potential to form functional OLGs *in vivo*, the tremor and survival rate were test in the subsequent experiment. After OPCs transplantation, the tremor was significantly decreased compared the untreated Shi mice (Figure 5C and Supplementary Videos 1, 2). As shown in Figure 5D, the survival rate of mice transplanted with OPCs was increased, with a range of 106–135 days. All PBS-treated Shi mice died, over a range of 90–125 days postnatally. These results indicated that BM-OPCs could successfully differentiate into functionally OLGs, induce remyelination, and increase survival rate in Shi mice. The experimental design overview for this protocol is shown in Figure 6.

## Discussion

Oligodendrocyte progenitor cells can be isolated from the SVZ of adult mammalian and expanded *in vitro* for producing mature OLGs (Chen et al., 2007). However, with this method, a little number of mature OLGs can be obtained. Recent studies showed that it is possible to differentiate OPCs from BM cells (Yang et al., 2010; Nazm Bojnordi et al., 2015). Indeed, bone marrow-derived OPCs showed distinct neural cell markers and morphology (Abbaszadeh et al., 2013). The possibility to get a high number of mature OLGs, starting from bone marrow, represents an accessible, and alternative manner respect to canonical methods in which OPCs are generated from SVZ-derived cells. OPCs transplantation either intravenous (i.v.) or i.c.v. has shown to promote remyelination *in vivo* (Sher et al., 2012; Wu et al., 2012); thus its therapeutic potential is relevant for demyelination disorders.

Currently, there are several methods for obtaining functional OLGs (Garcia-Leon and Verfaillie, 2016). Nazm Bojnordi et al. (2015) used BM stromal cells for producing functional Olig2 and O4^+^ cells. These OPCs successfully promoted the remyelination, in LPS-induced demyelination model; however, the efficiency was unclear. Further, OPCs isolation from ESCs or induced pluripotent stem cells (iPSCs), is an effective way for repairing and preventing the progression of demyelinating lesions (Douvaras et al., 2014; Thiruvalluvan et al., 2016). However, ESCs-derived OPCs may lack of stability and safety. It has been shown that NSCs possess less risk of potential tumorigenicity than ESCs or iPSCs (Zhao et al., 2015). The generation of OPCs from BM is consistent with what demonstrated so far (Yang et al., 2010; Hojjat-Allah et al., 2013; Nazm Bojnordi et al., 2015). Our method mainly consists of three steps, bone marrow-derived NSCs generation, NSCs-OPCs differentiation, and OLGs maturation. These data also focused on the derivation of OLGs from OPCs using assorted agents. In all of these steps, we use conditioned-media containing essential growth factors such as T3, Shh, IGF, and NT-3. We, also, use Noggin for negatively regulating bone morphogenetic protein (BMP) pathway, which blocked oligodendrocyte maturation (Najm et al., 2013). Although this protocol does not significantly improve the differentiation efficiency, compared with previous work, the timing is shorter (from 75 to 95 and 52 days) (Chen et al., 2007; Douvaras and Fossati, 2015). Our findings provide an alternative, fast strategy for obtaining functional OLGs from BM cells after just 40–50 days of differentiation. Here, we use the congenital dysmyelination model of Shi mice to show that BM-OPCs improved the mice survival. Our protocol can generate OPCs from BM-NSCs providing sufficient cell sources from autologous and inducing remyelination.

One generally OPCs generating strategies is that the cell need transduction of several TFs by lentiviral (Han et al., 2012; Najm et al., 2013). Virus-mediated gene delivery required higher biosafety levels and still need to be considered for clinical application (Capetian et al., 2016). In our protocol, we choose a well-established, and effective custom differentiation media system (Najm et al., 2013; Baskin et al., 2016). These differentiation media contain several growth factors (EGF, FGF, PDGF, Shh, Noggin, IGF, and NT-3) which used in different proliferation or differentiation stage were sufficient for inducing cell differentiation into mature OLGs. The manner of using growth factor is safer than viral or small molecules to treat cell or neurodegeneration disease (Tuszynski et al., 2015; De et al., 2017). The specificity of growth factors to recognize its target is better than small molecules especially in high dose (such as concentration > 10 μM) (Weiss et al., 2007). Also, when withdrawing the growth factors such as FGF, EGF et al., cell will cease to proliferate and avoid the risk of formation tumor cell. In a previous report, T3 was used as an inducer to differentiate into oligodendrocyte-like cells using autologous BM stromal cells (Kaka et al., 2012; Hojjat-Allah et al., 2013). Compared with their researches, we have demonstrated that mouse OLGs can be produced from bone marrow-derived cells *in vitro*, indicating that our method is more reliable. Together, this protocol indicates that our bone marrow-derived OPCs represent a simple, safe, valuable and promising cell-based therapy strategy for myelin repair and neurodegeneration disease.

## Ethics Statement

All experimental procedures and protocols are approved by the Animal Management and Committee of Shaanxi Normal University. At the same time, in accordance with the approved institutional guidelines and regulations.

## Author Contributions

YZ and XL conceived and designed the experiments. YZ, X-YL, JT, J-JH, and Z-QY carried out the experiments. TY, L-YJ, and XL analyzed the data. YZ, X-YL, GC, JT, and XL wrote the manuscript. AR and XL supervised the study and revised the manuscript. All authors read and approved the final manuscript.

## Conflict of Interest Statement

The authors declare that the research was conducted in the absence of any commercial or financial relationships that could be construed as a potential conflict of interest.
